# Metabarcoding the Antarctic Peninsula biodiversity using a multi-gene approach

**DOI:** 10.1038/s43705-022-00118-3

**Published:** 2022-04-13

**Authors:** V. G. Fonseca, A. Kirse, H. Giebner, B. J. Vause, T. Drago, D. M. Power, L. S. Peck, M. S. Clark

**Affiliations:** 1 Centre for Environment, Fisheries and Aquaculture Science (Cefas), Weymouth, UK; 2 Zoological Research Museum Alexander Koenig (ZFMK), Bonn, Germany; 3 British Antarctic Survey, Natural Environment Research Council, Cambridge, UK; 4 Portuguese Institute for Sea and Atmosphere (IPMA), Tavira, Portugal; 5 Institute Dom Luiz (IDL), University of Lisbon, Lisbon, Portugal; 6 Centre of Marine Sciences (CCMAR), Faro, Portugal

**Keywords:** Biodiversity, Community ecology, Ecosystem ecology

## Abstract

Marine sediment communities are major contributors to biogeochemical cycling and benthic ecosystem functioning, but they are poorly described, particularly in remote regions such as Antarctica. We analysed patterns and drivers of diversity in metazoan and prokaryotic benthic communities of the Antarctic Peninsula with metabarcoding approaches. Our results show that the combined use of mitochondrial Cox1, and 16S and 18S rRNA gene regions recovered more phyla, from metazoan to non-metazoan groups, and allowed correlation of possible interactions between kingdoms. This higher level of detection revealed dominance by the arthropods and not nematodes in the Antarctic benthos and further eukaryotic diversity was dominated by benthic protists: the world’s largest reservoir of marine diversity. The bacterial family Woeseiaceae was described for the first time in Antarctic sediments. Almost 50% of bacteria and 70% metazoan taxa were unique to each sampled site (high alpha diversity) and harboured unique features for local adaptation (niche-driven). The main abiotic drivers measured, shaping community structure were sediment organic matter, water content and mud. Biotic factors included the nematodes and the highly abundant bacterial fraction, placing protists as a possible bridge for between kingdom interactions. Meiofauna are proposed as sentinels for identifying anthropogenic-induced changes in Antarctic marine sediments.

## Introduction

Under the current global climate crisis, there is a pressing need to understand how changing environmental conditions impact future biodiversity, particularly in those areas experiencing rapid climate change. One such area is the Western Antarctic Peninsula (WAP). Over the past 50 years the seas around the WAP have experienced, and are still experiencing, very rapid rates of regional warming resulting in glacier retreat and reductions in annual sea-ice extent [1]. These changes drive habitat and biome shifts with potentially significant impacts for biodiversity and ecosystem functioning. Local consequences of warming are already evident in data from long-term marine monitoring studies, such as the regular oceanographic and biological observations carried out at Rothera Research Station on the Antarctic Peninsula. Observations include a reduction in sea-ice cover, leading to decreased water stratification, impacting the magnitude, duration and composition of phytoplankton blooms [2, 3]. Furthermore, the reduction in sea-ice appears to affect reproductive capacity of some benthic invertebrates and led to increased iceberg scour, with consequent destructive impacts on benthic communities [4, 5]. Whilst these studies have highlighted directional trends beyond inter-annual variability, they do not include analyses of marine sediment communities. This is a significant knowledge gap as these communities play key roles in food webs, biogeochemical cycling and contribute significantly to ocean health and functioning [6, 7].

The limited analyses of sediment meiofaunal biodiversity around Rothera demonstrated levels of biodiversity similar to temperate regions, with grain size as a major driver of diversity [8, 9]. Meiofauna are critical elements of marine ecosystems and macrofauna predation accounts for 75% of meiofaunal production transferred to higher trophic levels [10]. Since many macrofaunal larvae are a similar size to adult meiofauna they are potential meiofauna prey items, and this has implications for macrobenthic recruitment and higher-level community structures [11]. However, meiofauna are one part of sediment communities and bacteria typically dominate marine sediment biomass [12]. Benthic bacteria play a major role in the remineralization of organic matter and represent a significant store, and source of, carbon in our oceans [13]. This provides the living foundation for sediment ecosystems and biogeochemical cycling, with the dominant metabolic pathway resulting from a range of biotic and abiotic factors [14]. Bacteria rapidly metabolise dissolved organic matter to fuel growth and the resulting bacterial biomass is a major food source for larger organisms. Bacteria are also important in the processing of detrital material and faecal pellets, thus exerting a strong influence on the availability of key nutrients for primary producers [15]. In fact, detritivores derive more energy and nutrients from detritus-associated bacterial communities than from the detritus itself, a process often called ‘Trophic Upgrading’ [16].

There is strong interdependence between the micro- and meiofauna in sediments [17]. For example, bioperturbation by larger animals (e.g. nematode and bivalve burrowing) and cryptobioperturbation of surface sediments by meiofauna can redistribute nutrients and alter oxygen fluxes, creating fertile microniches for microbial communities [18]. Furthermore, mucus secretions by caudal and oesophageal glands of nematodes attract bacteria as food sources and as producers of organic matter [19] with nematode richness having a positive effect on microbial activity [20]. Thus, the different elements (i.e. size classes, life cycle, ecology) of marine sediment organisms should not be considered separately as they are inextricably linked. Such combined studies are particularly important as the micro- and meiobenthic fauna in sediments, respond rapidly to changing environmental conditions and have been proposed as efficient environmental sentinels [7, 21, 22].

Hence, the aim of this study was to address the paucity of data on Antarctic sediment communities and provide a baseline for future monitoring and understanding of how regional climate change impacts biogeographic shifts of microbial and meiofaunal assemblages in this region. We metabarcoded the mitochondrial cytochrome c oxidase subunit I (Cox1) and the ribosomal 16S and 18S (rRNA) gene region, to produce the first multi-gene analysis of shallow-water sediment microbial and meiofaunal biodiversity communities around Rothera Research Station on the Antarctic Peninsula. Biodiversity patchiness was characterised spatially across three sites, with high levels of endemicity, community composition correlated with environmental variables, such as sediment characteristics and associations between Eukaryotes and Bacteria are discussed.

## Material and methods

### Sample collection and processing

**Fig. 1:**
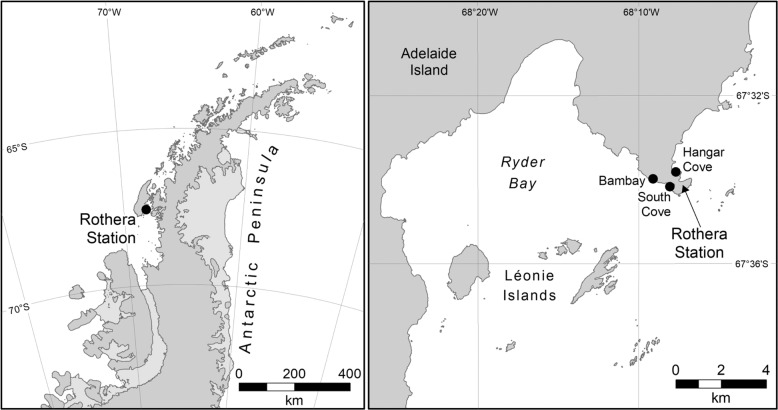
Sediment sampling locations in the Antarctic Peninsula. Location of Rothera Research Station on the Antarctic Peninsula and sample sites around Rothera (Hangar Cove, South Cove and Bambay).

During March 2013 sediment samples were collected in triplicate every 100 m at 15–20 m depth from three sites: Bambay (S67° 34.00333 W68° 8.955 and S67° 33.98167 W68° 9.245), South Cove (S67° 34.12167 W68° 7.95333 and S67° 34.23833 W68° 7.91833) and Hangar Cove (S67° 33.91333 W68° 7.81333 and S67° 33.82333 W68° 7.56) near the British Antarctic Survey Rothera Research Station, West Antarctic Peninsula (Fig. 1). The substratum in South Cove and Bambay is a mixture of bedrock and cobbles with patches of soft sediment, though Bambay is smaller in area than South Cove and patches of sediment are smaller in area. Conversely, Hangar Cove primarily has a base of compacted cobbles covered with a thin layer of fine sediment (2–3 cm) interspersed with patches of, deeper, fine sediment. In all three coves the thickness of the soft sediment ranges from 0 to ~20 cm with a gently sloping topography. The sediment in these coves is primarily silt and fine sand ranging from 0.06–0.1 mm [23] and all three bays receive sediment from glaciers at the head of the bay. During summer icebergs impact the benthic communities in these coves and in the winter the sea surface is covered by fast ice for 3 to 8 months [24]. From each location triplicate samples (total *N* = 9) were taken from undisturbed sediment, using a hand-held plastic corer; 8 cm diameter × 20 cm length and the collected sediments were stored in the cores at −80 °C until processing.

### DNA extraction from sediments

For each sample the top 10 cm of sediment was cut while still frozen, mixed with a sterile metal spatula for homogenisation and during this process any larger organisms detected were discarded from the 10 g of starting material used for DNA extraction. DNA extraction was performed using the PowerMax® Soil DNA Isolation Kit (MO-BIO) following the manufacturer’s instructions. DNA extracts were visualised by agarose gel electrophoresis, quantified using a NanoDrop2000 spectrophotometer and extracted DNA diluted to 10 ng/µl and stored at −20 °C for downstream molecular analysis.

### NGS libraries

Two ribosomal (16S and 18S rRNA) and the mitochondrial Cox1 gene were used in the PCR amplifications. The universal primers ‘TAReuk454FWD1’ (5ʹ-CCAGCASCYGCGGTAATTCC-3ʹ) and ‘TAReukREV3’ (5ʹ-ACTTTCGTTCTTGATYRA-3ʹ) were used to amplify ~400 bp of the 18S V4 region [25]. The forward 515Fw (5ʹ-GTGCCAGCMGCCGCGGTAA-3ʹ) and reverse 806Rv (5ʹ-GGACTACHVGGGTWTCTAAT-3ʹ) primers were used to amplify ~300 bp of the 16S V4 region, recommended for microbial biodiversity studies [26]. The mitochondrial Cox1 313 bp gene region was amplified using the primers forward ‘mlCOIintF’ (5ʹ GGWACWGGWTGAACWGTWTAYCCYCC-3ʹ) and reverse ‘jgHCO2198’ (5ʹ-TAAACTTCAGGGTGACCAAARAAYCA-3ʹ) [27]. All primer combinations were designed to include the Illumina MiSeq 8nt index-tags (i5/i7) and Adaptors (P5/P7). Each replicated sample site had these primer-adaptor tags to allow differentiation of sequenced samples. PCR amplification of the specified rRNA and Cox1 gene sequence was performed with a 2-step PCR approach using Pfu DNA polymerase (Promega). The first PCR involved 5 min denaturation at 95 °C, then 20 cycles with 30 s at 98 °C, 30 s at 50 °C (rRNA) or 45 °C (Cox1), 30 s at 72 °C and a final extension of 10 min at 72 °C. To add the Illumina index tag adaptors a second PCR was performed using the same conditions reported above but with 10 cycles and an annealing temperature of 55 °C. Negative controls were included for all amplification reactions. The second PCR products were visualised and purified from agarose gels (QIAquick Gel Extraction Kit, Qiagen) and quantified using an Agilent Bioanalyser. All PCR products were diluted to the same concentration, pooled to create one metagenetic sample/library and pair-end sequenced on a MiSeq platform using v2 Illumina chemistry (2 × 250bp).

### Meta-data analysis

The 16S, 18S and Cox1 raw sequences from the Illumina MiSeq platform were pre-filtered by removing primer sequences from the forward and reverse reads using the default settings in cutadapt version 1.18 [28]. Forward and reverse reads were trimmed simultaneously, and were discarded if either the forward or reverse read pair was not within an error tolerance of 10% over a minimum overlap of ten nucleotides. Trimmed sequences were analysed using the dada2 plugin within QIIME2 [29]. This plugin produces amplicon sequence variants (ASVs), resolving differences as small as a single nucleotide [29]. Filtered and chimera-free ASVs were then assigned a taxonomy using the ‘feature-classifier’ plugin and the ‘classify-consensus-blast command’ within QIIME2 [30]. Taxonomy assignment was performed three times using different identity thresholds (95%, 97%, and 99%). Optionally, all redundant BLAST matches were merged using the ‘filter-seqs’ and ‘collapse’ commands of the qiime2 ‘taxa’ plugin [29] to remove redundancy in the taxonomy assignments based on a specific taxonomic level (species). To identify taxon relative abundance and direct ecological comparisons, the sequencing data was normalised using the phyloseq R package [31], using the lowest sequencing depth per sample (62,322 reads for 18S, 118,465 for Cox1 and 241,418 for 16S) and randomly resampled reads (rarefy_even_depth) in order to equalise the sampling effort between samples [31] (Supplementary Information Figs. S1, S2, S3).

### Reference databases

For the 16S dataset, a pre-compiled QIIME2 compatible reference dataset from SILVA release 132 was used [32], which was made non-redundant by clustering at 99% similarity and linking majority-based taxonomy strings, resulting in a total reference database of 369,953 sequences. For the 18S and mitochondrial Cox1 markers, reference sequences and taxonomies were downloaded from GenBank [33] and brought into a QIIME2 compatible format using in-house Perl scripts. During this process downloaded sequences were length filtered (18S: 500–2500 bp; Cox1: 500–2000 bp) and further processed, by discarding entries from environmental sequences, with missing taxonomic assignments from phylum to genus level. The resulting 18S reference database consisted of 669,693 entries, whilst the Cox1 reference database encompassed 2,857,567 entries.

*Sediment characterisation:* All sediment samples were characterised as follows: *Water content:* Sediment water content was determined before and after drying at 40 °C. Water content (%) was determined as water weight (Ww) divided by dry sediment weight (Wds) and multiplied by 100 [(*W* = Ww/Wds)*100]. *Grain size:* Sediment samples were submitted to wet separation of mud (silt and clay) from the sand fraction with a 63 µm sieve. The mud fraction (<63 µm) was recovered by sequential decantation of percolated material, which was dried and weighed. Sediments retained in the 63 µm sieve were subsequently dried and passed through a further sieve stack in a vibratory sieve shaker (5 min, amplitude 50%), to quantify the coarser fractions. The classes analysed, adapted from Wentworth 1922 [34] were: coarse gravel (>8 mm), medium gravel (8–4 mm), fine gravel (4–2 mm), very coarse sand (2–1 mm), coarse sand (1–500 µm) medium sand (500–250 µm), fine sand (250-125 µm) and very fine sand (125–63 µm). The contribution of the distinct fractions was calculated as a percentage. *LOI (loss on ignition):* The organic matter content of sediments was quantified through LOI [35]. About 200 mg of dry sediment, ground with a mortar and pestle, was burned at 450 °C for 2 h (ash weight). The organic matter content (ash-free dry weight) was estimated by the ratio: [(dry weight) − (ash weight)] × 100/ (dry weight). *Carbonates:* Carbonate content was determined using a calcimeter (Model 08.53, Eijkelkamp, Netherlands) which measures the volume of carbon dioxide produced during the reaction of the sample with hydrochloric acid [36]. The carbonate content was expressed as equivalent calcium carbonate content.

### Diversity and community analysis

To analyse meiobenthic community (di) similarities among sites, a similarity profile (‘SIMPROF’) permutation test was performed and significant differences in community assemblages, were tested using a permutational multivariate analysis of variance (‘PERMANOVA’). Analyses were based on Sørensen’s similarity coefficient on untransformed data of an ASV presence/absence matrix over the 9 sampled sites, with 1000 permutations. To investigate associations between community composition and environmental variables (sediment parameters) a BIOENV (‘biota-environment) analysis was conducted using Spearman’s rank correlation method. The BIOENV analysis searches for the best explanatory variables between meiofaunal communities and the abiotic parameters measured. Six environmental sediment related parameters were tested, as percentages of: water content, carbonates, organic matter, gravel (2–8 mm), sand (<1 mm) and mud (<63 µM). To further test and visualise the relationships between the environmental variables and community composition a principal Coordinates Analysis (PCoA) was performed using the software Primer 6 (v6.1.16) to explore community dissimilarities between samples (beta diversity). Phylum-specific sample-size-based R/E curves with extrapolations of Hill numbers for presence-absence data were prepared using the R-package iNEXT at default settings (40 knots, 95% confidence intervals generated by the bootstrap procedure (50 bootstraps)) (Supplementary Fig. S4). To test for phylum specific associations a non-parametric Mantel-type test (‘RELATE’) based on phylum community composition (presence–absence data) was performed using Primer 6 (v6.1.16). Rarefaction and accumulation curves (sequencing depth and sampling effort) were performed on presence–absence matrices for each marker using a 99% sequence identity threshold in the R package iNEXT [36, 37] (Supplementary Fig. S5).

## Results

**Fig. 2:**
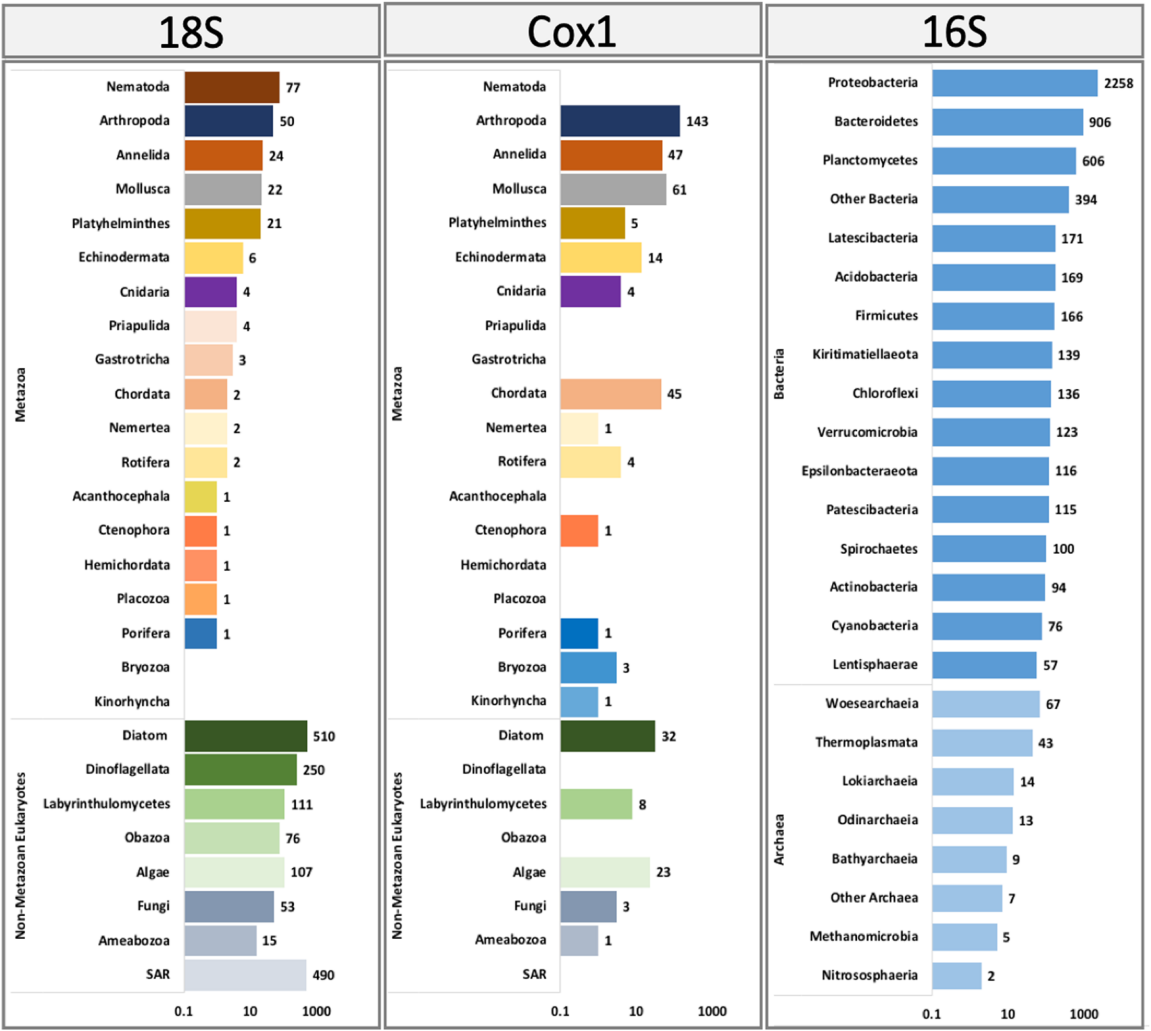
Taxonomy assignments obtained with the 18S rRNA, Cox1 and 16S rRNA found in Rothera, West Antarctic Peninsula. Main metazoan and non-metazoan taxa assigned to 18S and Cox1 are shown as well as the main bacteria and Archaea groups assigned to the 16S. The scale was transformed logarithmically and the total number of ASVs are indicated for each taxa.

**Fig. 3:**
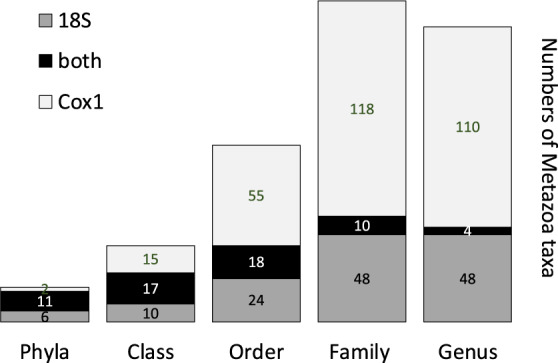
Number of Metazoa taxa. Unique to each taxonomic ranking for Cox1 and 18S markers and shared between markers.

The normalised sequencing data did not affect the diversity patterns (Supplementary Figs. S1, S2, S3) and overlying conclusions, thus the results presented are based on non-normalised raw datasets analysed with dada2 in QIIME2. After filtering and quality control, the mean range of total raw-reads per sample was between 1.9M (18S) to ca. 2.5M (16S, Cox1). The analyses were run individually for each gene and after denoising (trimming, quality filter, chimera removal) the mean number of chimera-free reads per sample was 638,144 (18S), 922, 186 (16S) and 1,073,044 (Cox1) (Supplementary Table S1), which were then assembled into amplicon sequence variants (ASVs). Taxonomic assignments using 16S retrieved a total of 5791 prokaryotic ASVs, of which 5630 were bacteria and 161 were Archaea ASVs. The three most abundant bacterial groups comprised the Proteobacteria, Bacteroidetes and Planctomycetes (Fig. 2). The more ASV rich metazoan groups identified by 18S were the Nematoda (77 ASVs) followed by the Arthropoda (50 ASVs), Annelida (24 ASVs), Mollusca (22 ASVs) and Platyhelminthes (21 ASVs). The Arthropoda were the dominant group (143 ASVs) identified by Cox1 followed by the Mollusca (61 ASVs), Annelida (47 ASVs) and Echinodermata (14 ASVs), other metazoan groups were identified by both markers (18S and Cox1) with less than ten ASVs (Fig. 2). Non-metazoan groups mainly identified with 18S were dominated by diatoms (510 ASVs) followed by the group SAR (Stramenopiles, Alveolata, Rhizaria) (490 ASVs). Non-metazoan groups further identified using 18S were the dinoflagellates (250 ASVs), Labyrinhulomycetes (protists) (111 ASVs), algae (107 ASVs) and Obazoa (a clade consisting of the Opisthokonta, Apusomonadida and Breviatea) (76 ASVs). The Cox1 gene also identified non-metazoan eukaryotes including diatoms (32 ASVs), algae (23 ASVs) and others (<10 ASVs) (Fig. 2). Additionally, eleven (55%) of the phyla found were identified by both the Cox1 and 18S markers but only 10 (6%) and 4 (3%) of the family and genus were common to both markers (Fig. 3).

**Fig. 4:**
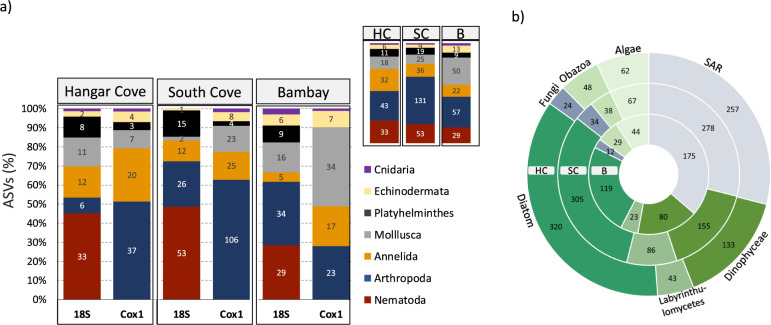
Eukaryotic diversity found in Hangar Cove, South Cove and Bambay. ASVs proportions for the most dominant Metazoa (**a**) and non-metazoa (**b**) found in the 3 sampling locations in the Antarctic Peninsula using 18 S and Cox1 genes. **a** unique ASVs proportions per marker and for the combined markers per sample (top right-hand graph). **b** non-metazoan ASVs obtained only with 18S per sampling site. HC-Hangar Cove outer circle, SC-South Cove middle circle, B-Bombay inner circle. Number of ASVs are shown in numbers.

Both markers (Cox1 and 18S) showed similar meiobenthic structure for the most dominant (ASV rich) meiobenthic metazoans. In general, the most abundant phyla were the Arthropoda, and Nematoda, followed by the Annelida, Mollusca and Platyhelminthes (Fig. 4a, Supplementary Fig. S1). The Cox1 amplicon sequencing results for South Cove identified double the number of Arthopoda ASVs (106) compared to the Nematoda (53 ASVs). The Annelida were more abundant in Hangar Cove and South Cove with ~25 ASVs whereas in Bambay there were fewer identifications (17 Annelida ASVs). The Mollusca were more abundant in Bambay (34 ASVs) when compared to the other locations (11–23 ASVs). Platyhelminthes, Echinodermata and Cnidaria were present in all locations but with fewer ASVs (Fig. 4a, Supplementary Information Fig. S1). The 18S gene also identified non-metazoans, listed in overall descending numbers of ASVs, the diatoms, Dinophyceae, Labyrinthulomycetes, SAR (Stramenopiles, Alveolata, Rhizaria), Obazoa, algae, fungi and Chlorophyta (Fig. 4b). In general, for all non-metazoans the Bambay site always had around half the ASVs compared with Hangar and South Coves whereby diatoms and SARs were the most dominant with ca. 300 and 250 ASVs, respectively (Fig. 4b, Supplementary Information Fig. S1).

**Fig. 5:**
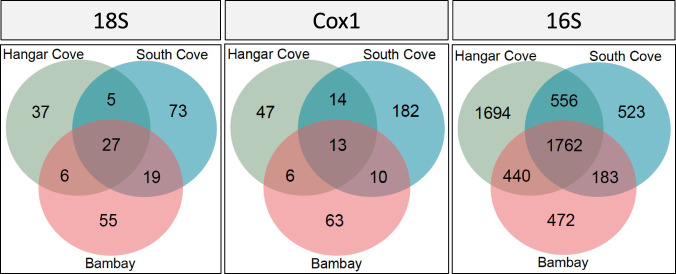
Shared and unique ASVs between the three sampling locations in the Antarctic Peninsula. The Venn diagrams present the number of site unique ASVs, those shared between two sites and those shared between the three sites for the 18S and Cox1 (eukaryotes) and 16S (prokaryotes) genes.

The analysis of metazoan ASVs found per sampling location showed that only 4% (Cox1) to 12% (18S) of the ASVs were common to all sampling sites (Fig. 5). Independently of the marker, the number of unique ASVs in each site was higher (from 37 to 182) when compared with shared ASVs (from 5 to 73). More unique metazoans were found at South Cove (127 ASVs), followed by Hangar Cove (60 ASVs) and Bambay (59 ASVs). Bambay and South Cove shared more metazoan ASVs than with Hangar Cove. The analysis of 16S showed that 31.3% (1762 ASVs) of bacterial ASVs were common to all locations, with 48% of bacterial ASVs unique to a particular site, from which ca. 30% (1694 ASVs) were exclusively found in Hangar Cove and the remaining 9% (523 ASVs) and 8.4% (472 ASVs) unique to South Cove and Bambay, respectively (Fig. 5). The remaining 21% of bacterial ASVs were shared between two sites only. Hangar Cove shared more ASVs with South Cove (556 ASVs) and Bambay (440 ASVs) and the least shared numbers of bacteria were between Bambay and South Cove (183 ASVs). From a total of 15 main bacterial phyla, three were most dominant, the Proteobacteria, Bacteroidetes and Planctomycetes, with more than 500 ASVs in at least one sampling location. Higher numbers of bacteria for most phyla were always identified in Hangar Cove when compared to South Cove and Bambay. Other bacterial phyla were found with ca. 100 ASVs per sampling location, which included the Latescibateria, Acidobacteria, Firmicutes, Kiritimatieallaeota and Chloroflexi. The remaining bacterial phyla identified had less than 100 ASVs (Supplementary Fig. S2).

Community assemblages using BIOENV analysis showed that the combined effect of most granulometric variables measured accounted for much of the variance observed and were significantly correlated with community structure for Eukaryotes in general (rho > 0.70, *P* < 0.01) (Table 1). The only granulometric parameter that had no effect on community structure was gravel particle size (rho = 0). Particularly high and significant correlations (rho = 0.75, *P* < 0.001) were obtained between metazoan assemblages and sediment water content, organic matter (OM) and mud. The Nematoda were the only metazoan group with strong correlations (rho = 0.73) with water content, carbonates and OM. Within the non-metazoa the bacteria had significantly high correlations (rho > 0.70, *P* < 0.001) with all granulometric parameters, with the exception of gravel. In particular, the phylum Planctomycetes and the group SARs were very highly correlated (rho = 0.73, *P* < 0.001) with water content and mud, whilst the SARs also showed additional effects of carbonates and sand (Table 1).

**Table 1:** Correlations found between the combined effect of environmental parameters and community composition for the main phyla. BIOENV analysis using Bray-Curtis index and BEST spearman correlation (rho) with 1000 permutations.

rho values shown in grey gradients: dark grey (0.75 < rho < 0.73), grey (0.73 < rho < 0.71), light grey (0.71 < rho < 0.65) to white (<0.5). Water content, carbonates and organic matter (OM) are measures of percentage. Arthropoda and Annelida rho values from Cox1 presence–absence matrix. Non-significant and low rho values were excluded from the analysis (e.g. Gravel rho = 0, Archaea, Obazoa, Mollusca rho < 0.4; *p* > 0.05).

**Fig. 6:**
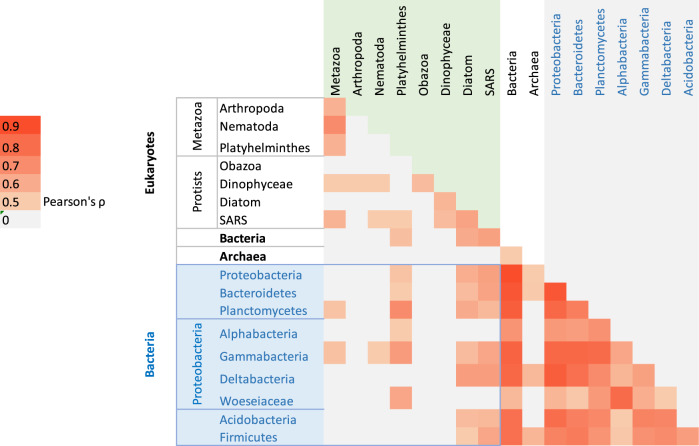
Heatmap showing significant associations between the main groups of Eukaryotes, Bacteria and Archaea. Pearson correlation rho values range from 0 to 0.9, grey to dark red, respectively. Associations between prokaryotes (Bacteria/Archaea) and eukaryotes are highlighted in the blue shaded box and with blue letters. Associations within Eukaryotes are shaded in green and within Bacteria/Archaea are shaded in grey. Only dominant phyla that exhibited *p* < 0.05 and rho > 0.5 are represented. rho values of zero comprise 0 < rho < 0.45.

Overall, there was a significant positive association between Bacteria groups, with minimum associations observed between Woseiaceae (rho = 0.57) and maximum associations between Proteobacteria (rho = 0.97), with average significant associations between all Bacteria groups of 0.83 (*p* < 0.05). Associations within eukaryotes were weaker, ranging from 0.5 between diatoms and Metazoa and 0.74 between Nematoda and Metazoa, with an average rho of 0.55 (*p* < 0.05). The eukaryotic phyla with the highest associations with Bacteria were the Platyhelminthes, diatoms and SARS. In particular, the Planctomycetes (rho = 0.74), Gammabacteria (rho = 0.68) and Woeseiaceae (rho = 0.64) were associated with the Platyhelminthes and also with diatoms and SARS (Fig. 6). Phylum-specific and sampling accumulation curves did not reach an asymptote showing incomplete sampling effort (Figures Sp and Sc) but rarefaction curves for all markers and samples reached an asymptote indicative of sufficient sequencing depth (Supplementary Figs. S4, S5).

**Fig. 7:**
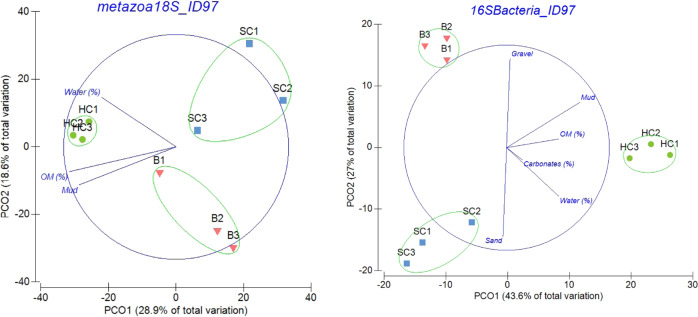
Principal Coordinate analysis based on similarity profiles (green line > 70% similarity) between samples for Metazoan (18S) and Bacteria (16S). Analysis was based on Sorensen similarity coefficient using a presence-absence matrix. Environmental variables that showed the greatest individual effect (BIOENV, Pearson rho > 0.45) on each phyla community structure are also represented (blue lines). SC-South Cove; HC-Hangar Cove; B- Bambay.

Beta diversity analysis revealed a clear separation of sample site according to community composition with more than 70% similarity clusters between sites. These similarity profiles along the PCoA axis explained 33.7% of the variation found for Eukaryotes, 28.9% for the Metazoa and 43.6% for the bacteria community structure (Fig. 7, Supplementary Information Fig. S6). Other similarity profiles analysed for eukaryotes, nematodes, diatoms, SARs, planctomycetes, Bacteria and Archaea also showed distinct location clusters with up to 45.1% of the variation found explained by the analysis. For the Nematoda, South Cove and Bambay communities overlapped and Hangar Cove formed a distinct cluster (Supplementary Fig. S6).

## Discussion

This study clearly shows the importance of using multiple gene markers in biodiversity surveys, with little overlap at the family and genus level between Cox1 and 18S. The use of both makers allowed identification across broader taxonomic groups when using the 18S, and better species-level resolution when using the Cox1, the latter is particularly good for targeting arthropods [38, 39] (Figs. 3, 4). Furthermore, the use of 16S enabled the investigation of possible interactions between kingdoms. The use of several markers provides better estimates of biodiversity [40] and improve taxonomic resolution by up to 10% [41]. This multi-barcode approach placed arthropods as the most dominant organisms of the Antarctic benthos, contrary to what was previously found when using just the 18S [8]. Likewise, the Platyhelminthes previously listed as the third most dominant taxon [8], when adding more power to the genetic analysis, annelids and molluscs were promoted to the third and fourth most dominant phyla in the Antarctic soft seabed benthos. Nonetheless, finding fewer Platyhelminthes might be due to sampling issues (e.g. crypsis and delicate body structures) and primer bias towards some taxa [42, 43]. Although, these new findings are not surprising since more markers will uncover more diversity and allow higher resolution levels [41], it acknowledges the importance of such approaches to better reflect local diversity levels. The phylum and sample (Supplementary Figs. S5 and S2.2) accumulation curves further evidence that the sampled Antarctic habitats are yet to fully describe local eukaryotic diversity and that more biological replicates and additional markers would be needed (including other gene regions if budget is not a constraint). However, this study adds new facts to the existing knowledge on Antarctic meiobenthic biodiversity in shallow waters, placing arthropods as the most dominant in the meiobenthos, followed by nematodes and annelids [8, 9, 44, 45]. These findings are fundamental to identifying key species in the marine benthos, understanding trophic relationships and evaluating ecosystem dynamics that globally sustain many other marine life forms [46].

The Antarctic marine benthos harbours diverse communities with high levels of endemicity, including non-metazoan microorganisms [8]. The high prevalence of diatoms and SARs (stramenopiles, alveolates and rhizaria) found in this study was no exception. Diatoms are part of the SAR super group ‘stramenopiles with chloroplasts’ but were analysed separately since they are one of the most important autotrophs in marine habitats [47]. Benthic diatoms play key roles in biogeochemical cycles and can also be the main food source of nematode and copepod species [47, 48] and thus, are very species rich and most widespread across several habitats. Although benthic protists have received little attention compared to their planktonic counterparts, the SAR super group identified may well be part of one of the world’s largest reservoirs of marine diversity [49]. The benthic protists are thought to be more diverse than planktonic protist communities [49] and in this study we found ~1000 benthic protist ASVs, which were poorly annotated and most are still not formally described (Supplementary Fig. S7).

### Alpha diversity

Previously we showed that meiobenthic communities tend to have high alpha diversity in both temperate and Antarctic regions [8, 9, 50]. This study now extends these observations across kingdoms, from metazoans to bacteria. Despite the fact that microorganisms are reported as cosmopolitan species [51] the high levels of endemicity found in Antarctic meiobenthic communities suggests that these are partly niche-driven [8, 45, 52] (Fig. 5). Although the three sites are relatively close together (within 1–2 km) they all exhibited significant diversity in both metazoan and bacterial communities. This is the result of a complex mix of seabed topography, ocean currents and freshwater run-off from glaciers, producing individual and specialised habitats within each cove (Lloyd Peck, pers. obs.). South Cove and Bambay are most similar in terms of seabed topography, both comprising unconsolidated mixed aggregates of boulders, pebbles and sediment, with the sediment present in lenses over solid rock, whereas Hangar Cove consists of layers of sediment including an anoxic layer [23, 24]. Hence, it was not surprising to find higher metazoan diversity in South Cove compared with Hangar Cove. The mixed aggregate nature of the South Cove seabed results in many small protected areas between boulders and stones, which can harbour the Metazoa, whereas the relatively homogeneous muddy areas in Hangar Cove are more suitable for bacteria. Here, the most dominant bacteria were Proteobacteria, followed by Bacteroidetes and Planctomycetes. Benthic bacterial assemblages in sediment show typical dominance by Proteobacteria, which are characteristic of deep-sea microbiomes [53], but also dominate the water column in the Antarctica, comprising the vast and vital bacterioplankton community [54, 55]. As also found here for sediments, the bacteria groups Alphaproteobacteria and Gammaproteobacteria dominate and are key colonisers of Antarctic surface and deep marine waters [54, 56, 57] which can thrive in areas with limited resources [58]. Other Proteobacteria such as the Planctomycetes contribute to benthic communities by increasing organic-matter in depleted subsurface sediments [59]. Although, both South Cove and Bambay are subject to similar currents, Hangar Cove experiences a different regime, with all three bays receiving different levels of fresh water run-off from glaciers. Habitat characteristics seem to be key for benthic and bacterial community structure. For example, South Cove, without a glacier near-by had more Metazoa than Bambay, which receives some glacial melt water from Reptile Ridge, whilst Hangar Cove on the opposite side of Rothera Point runs alongside the Wormald Ice Piedmont. This glacier is in retreat with regular melting and ice calving events and hence the fauna in Hangar Cove will experience regular and often considerable freshwater input. Nonetheless, meiofaunal organisms are clearly well adapted for living near to melting ice with osmoregulatory mechanisms that allow them to survive across a wide spectrum of salinities [60].

### What drives Antarctic benthic community structure?

Significant endemism is characteristic of polar ocean meiofauna [8, 61]. The environmental parameters measured that showed the highest correlation with Metazoa were water content and organic matter (Table 1). Sediment chemistry seems to be one of the drivers of meiobenthic communities along with the availability and composition of organic matter [62–64]. Usually, marine metazoan diversity is highest in wet, organic matter rich sediments [65] and both parameters showed the highest correlation across kingdoms, being two of the main drivers associated with microbial community structure (eukaryotes and prokaryotes) (Table 1). Similarly, Antarctic terrestrial bacteria were also reported to be quite site-specific and most affected by soil physical factors such as grain size and moisture [66]. Sediment grain size was also correlated with microbial community assemblages with the exception of gravel, which showed no correlation with any taxa. Most microbial benthic organisms live attached to sediment grains [67] and each sediment particle has different microenvironments that support several microbial taxa [68]. Ecological grouping in nematodes is often related to substrate type and feeding mode (e.g. microvores, predators) [69] and previous studies have further corroborated associations between sediment granulometry at local scales [70–72]. Usually coarser sediment like gravel, with larger pockets between particles can harbour rotifers and copepods, whereas finer sediments like muds are mainly occupied by nematodes and oligochaetes [69] but since the latter were dominant this could explain the absence of a correlation with gravel. This trend persisted within the Bacteria group, and bacteria more commonly found in coarser sediments were not identified in this study (e.g. *Gemmatimonadales)* whereas the most abundant taxa (e.g. Proteobacteria, Planctomycetes and Bacteroidetes) usually dominate muddy sediments, mostly comprised of fine silts and clay [68].

In general, seawater temperature can have the greatest impact on the diversity, abundance and structure of marine meiobenthic communities [50], and together with nutrient availability and chlorophyll-a it can impact significantly prokaryotic production and diversity [55, 73]. In the Antarctic Peninsula coastal systems, abiotic factors such as seawater temperature and oxygen availability are described as one of the drivers of eukaryotic community structure, whereas silicates and salinity tend to be more important for bacteria [57]. Local drivers of marine distributions in Antarctic benthic metazoa and marine bacteria are reported to be the result of many factors such as food availability, life-cycle strategies, season, depth, glaciers melting, substrate physical and chemical properties [8, 54, 74–76]. Among these factors, availability of organic matter and water content within the sediment, but also mud, had the highest correlations and seemed to have a strong effect shaping Antarctic benthic communities, both for bacteria and metazoans. Some authors suggest that organic matter is the main driver of Antarctic benthic community structure [74], and in fact it is also determinant in shaping prokaryotic communities in surface waters of the Southern Ocean [54]. Notably, Oztruk et al. [55] identified organic matter as one of the key factors shaping community structure in heterotrophs.

### Key phyla driving meiobenthic community structure

Associations between the main taxa were the highest within bacterial groups showing how intricate relationships between microorganisms can be, especially for the Proteobacteria, Bacteroidetes and Planctomycetes. The Proteobacteria found in this study, dominated by the Gammabacteria had the highest correlations with Metazoa, namely with Platyhelminthes and nematodes (Fig. 6). These groups of bacteria are dominant in several marine habitats, including sediments in the Western Antarctic Peninsula [74] and the Antarctic Polar Front [77]. The microbial diversity in the Southern Ocean increases with depth [55] and Proteobacteria and Bacteroidetes seem to dominate both surface waters [55–57] and sediments [this study, 53]. Bacteria that dominate the benthos are crucial for driving such communities, not only as decomposers but also as a food source for meiofauna [78]. While there is a consensus that Proteobacteria, Bacteroidetes and Planctomycetes thrive in marine sediments [79], the role of lower taxonomic bacteria is still unknown, especially if they are part of the ‘core microbiome [80]. In this regard, we describe here for the first time in the Antarctic Peninsula, the presence of the bacterial Woeseiaceae family. The Woeseiaceae have recently been highlighted due to their role in N-cycling [81, 82] and potential reduction of nitrous oxide (N_2_O) into the atmosphere [81]. Platyhelminthes were the only metazoan group showing significant correlations with the Woeseiaceae, perhaps due to their physiology (e.g. soft body, diffusion to breathe etc.). The Woeseiaceae have a widespread distribution and it is possible that they could have significant implications for N_2_O emissions from sediments [82]. Together with Platyhelminthes, they could potentially be used as sentinels of marine sediment changes, identifying shifts in biogeochemical cycles.

Within the meiofauna, nematodes have been shown to be inversely correlated with Platyhelminthes [70]. Although not observed in this study, nematodes were one of the main drivers of meiobenthic community structure since they showed the highest overall association values with the metazoa. Similarly, within the eukaryotic groups, diatoms and SARs can be the food of choice for most meiobenthic metazoans [83]. Benthic protists, one of the world’s largest reservoirs of marine diversity, are the primary bacterivores in most aquatic systems [84] and could be the missing link between meiofauna–bacteria interactions as described in other habitats like Red Sea sediments [78]. The dominance of the microbial fraction and its high association with metazoan communities emphasises their vital role in marine ecosystems.

### Local adaptation driving niche-specific metazoan and bacterial taxa

Overall PCOA explained up to 45% of community structure, suggesting that almost half the microbial patterns identified are factual despite incomplete sampling. Such incompleteness shows once more the immense benthic diversity of the Antarctic [8, 85, 86] and emphasises the importance of having enough biological replicates. The community profiles for bacteria and eukaryotes, including Metazoa were markedly dissimilar between locations. Although there was very little distance between the sampling sites, each site had distinct topographies promoting high levels of local diversity where 50% bacteria and 70% Metazoa were unique to each site. Each site also showed very clear separation in terms of community composition for both Bacteria and Metazoa, whereby each location clustered independently by 70% similarity, showing that such habitats harbour unique features for microbial communities (Fig. 7). Both Bacteria and Metazoa clearly adapt at the very local level (niche driven) observed by high ASV numbers per location, concomitant to species self-assembling into clusters based on their niche requirements [87]. A recent meiofaunal metacommunity analysis suggested that niche-based processes were the main structuring mechanism in both meiofauna and bacteria [88, 89]. This was substantiated in our study by the high alpha diversity found across kingdoms in each location. Both bacteria and eukaryotes were strongly associated with biotic and abiotic factors further supporting the species sorting paradigm whereby at local scales species occur in specific habitats where conditions are favourable [87, 89]. Considering that Antarctica contains some of the strongest environmental gradients on the planet [90], smaller spatial scale studies show how abiotic and biotic factors dictate diversity at habitat and community level [86]. This also highlights the high possibility for local extinctions and the importance of implementing strategies of site-specific management, as these will be key for sustaining unique levels of biodiversity and habitats in Antarctica.

## Supplementary information

Supplementary information

## Data Availability

The raw data from this study are available through NCBI’s Sequence Read Archive under Project Accession Number SUB8868490 (Bioproject PRJNA692247).
