# Supplementary material for: Metabarcoding the Antarctic Peninsula biodiversity using a multi-gene approach
Source: ISME Commun. 2022 Apr 13;2:37. doi: 10.1038/s43705-022-00118-3 (PMC9723778; doi:10.1038/s43705-022-00118-3)
Supplement: Supplementary file 1 — Supplementary information [file 43705_2022_118_MOESM1_ESM.pdf]

Fonseca VG, Kirse A, Hendrik G, Vause BJ, Drago T, Power DM, Peck LS and Clark MS. Metabarcoding the Antarctic Peninsula biodiversity: life under ice using a multi-gene approach.

Supplementary Information

Supplementary Table S1- Summary of *in silico* steps and read numbers for each gene region (16S, 18S rRNA and Cox1) per sample sample, using dada2 in QIIME2. Amplicon sequence variants (ASVs) taxonomically assigned using GenBank (18S and Cox1) and SILVA (16S) at 95%, 97% and 99% sequence identity BLAST match. Totals, mean and value ranges are highlighted in bold, yellow and blue for each step used, respectively.

**COI**	raw-reads	Trimmed read pairs	Quality-filtered read pairs	Merged read pairs	Chimera-free sequences	BlastID 95	BlastID 97	BlastID 99	All ASVs
Sample1	3610582	1594906	1505882	1498929	1482637	100	71	55	4875
Sample2	2159300	1010593	981198	976352	969540	71	55	41	2729
Sample3	1780044	790224	748956	743589	737252	56	34	23	1509
Sample4	3764112	1804004	1747986	1738499	1724209	159	108	76	4734
Sample5	4481442	2167416	2105554	2094811	2070336	233	175	125	4329
Sample6	3159366	1497456	1449845	1442204	1425235	140	101	66	4228
Sample7	1854074	904200	886683	883384	876693	59	52	45	847
Sample8	609322	265560	256556	254318	253027	70	53	37	1865
Sample9	347500	126797	120323	118935	118465	48	34	23	1616
**Total**	**21765742**	**10161156**	**9802983**	**9751021**	**9657394**	**544**	**407**	**301**	**14594**
Mean	2,418,416	1,129,017	1,089,220	1,083,447	1,073,044	104	76	55	2,970
range of values	4,133,942	2,040,619	1,985,231	1,975,876	1,951,871	185	141	102	4,028

**18S**	raw-reads	Trimmed read pairs	Quality-filtered read pairs	Merged read pairs	Chimera-free sequences	BlastID 95	BlastID 97	BlastID 99	All ASVs
Sample1	1153996	417276	390160	385899	382111	598	448	216	1298
Sample2	254770	70187	64409	63155	62322	269	200	103	510
Sample3	2990804	1207079	1085284	1079709	1050435	347	259	129	665
Sample4	4011200	1453165	1359586	1334122	1200328	1118	867	300	2103
Sample5	1944162	704054	656117	616689	580215	776	589	220	1420
Sample6	1655840	431352	386578	380650	356763	495	391	168	812
Sample7	1557808	652066	595968	591857	566416	338	273	136	551
Sample8	1686888	708291	654075	637086	588616	663	501	229	1252
Sample9	2629368	1099106	1028182	1010135	956089	1126	861	372	2008
**Total**	**17884836**	**6742576**	**6220359**	**6099302**	**5743295**	**2538**	**1863**	**711**	**5110**
Mean	1,987,204	749,175	691,151	677,700	638,144	637	488	208	1,180
range of values	3,756,430	1,382,978	1,295,177	1,270,967	1,138,006	857	667	269	1,593

**16S**	raw-reads	Trimmed read pairs	Quality-filtered read pairs	Merged read pairs	Chimera-free sequences	BlastID 95	BlastID 97	BlastID 99	All ASVs
Sample1	6846188	2674657	2603865	2555635	2428921	NA	3735	1998	7575
Sample2	5544208	2027813	1977631	1942785	1831767	NA	3362	1910	6144
Sample3	5457578	1601079	1544382	1508137	1437065	NA	3156	1736	5895
Sample4	1672890	743348	724240	697058	648270	NA	2050	1261	3318
Sample5	1859662	697761	679148	658666	619849	NA	2543	1500	4437
Sample6	1103820	444641	432608	419648	399040	NA	1719	1063	2963
Sample7	1299328	494079	477522	456140	434813	NA	2291	1366	3719
Sample8	918942	292235	280393	268994	258534	NA	2045	1228	3259
Sample9	955188	272095	261707	251107	241418	NA	1906	1183	2834
**Total**	**25657804**	**9247708**	**8981496**	**8758170**	**8299677**	NA	**5791**	**2896**	**13250**
Mean	2,850,867	1,027,523	997,944	973,130	922,186	NA	2,534	1,472	4,460
range of values	5,927,246	2,402,562	2,342,158	2,304,528	2,187,503	0	2,016	935	4,741

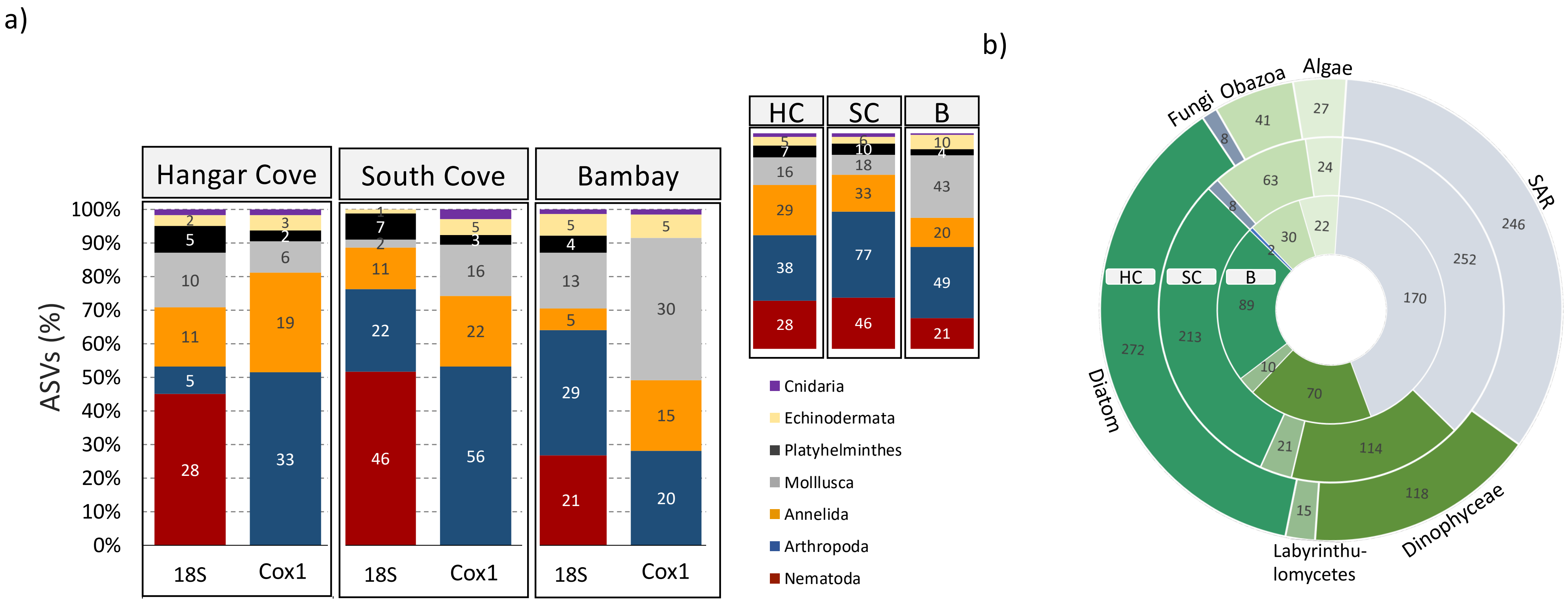

Supplementary Figure S1- Normalized ASVs for the most dominant Metazoa (a) and **non-metazoa** (b) found in the 3 sampling locations in the Antarctic Peninsula using 18S rRNA and Cox1 gene sequence. a) unique ASVS proportions per marker and for the combined markers per sample (top right-hand graph). b) non-metazoan ASVs obtained only with 18S per sampling site. HC-Hangar Cove outer circle, SC-South Cove middle circle, B-Bombay inner circle. Number of ASVs are shown in numbers.

Fonseca VG, Kirse A, Hendrik G,Vause BJ, Drago T, Power DM, Peck LS and Clark MS. Metabarcoding the Antarctic Peninsula biodiversity: life under ice using a multi-gene approach.

Supplementary Information

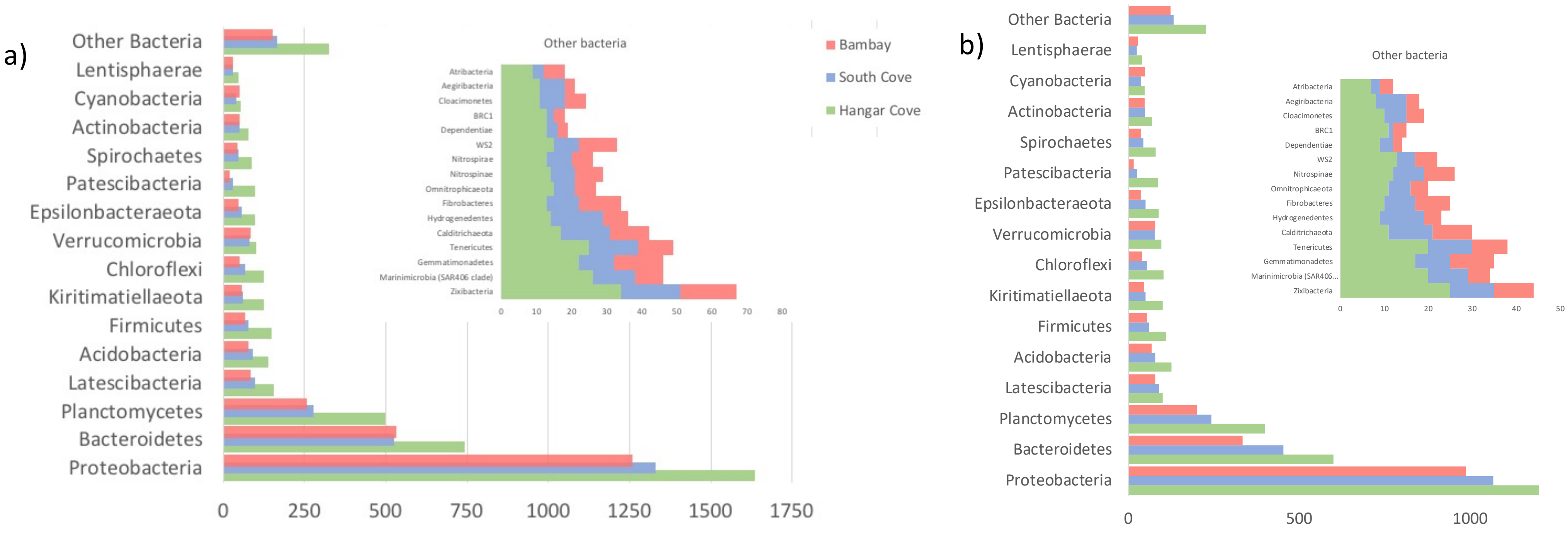

Supplementary Figure S2- ASVs numbers for bacteria taxa in the three sampling locations in the Antarctic Peninsula using the 16S rRNA gene region. a) non-normalized and b) normalized dataset using phyloseq.

Fonseca VG, Kirse A, Hendrik G,Vause BJ, Drago T, Power DM, Peck LS and Clark MS. Metabarcoding the Antarctic Peninsula biodiversity: life under ice using a multi-gene approach.

Supplementary Information

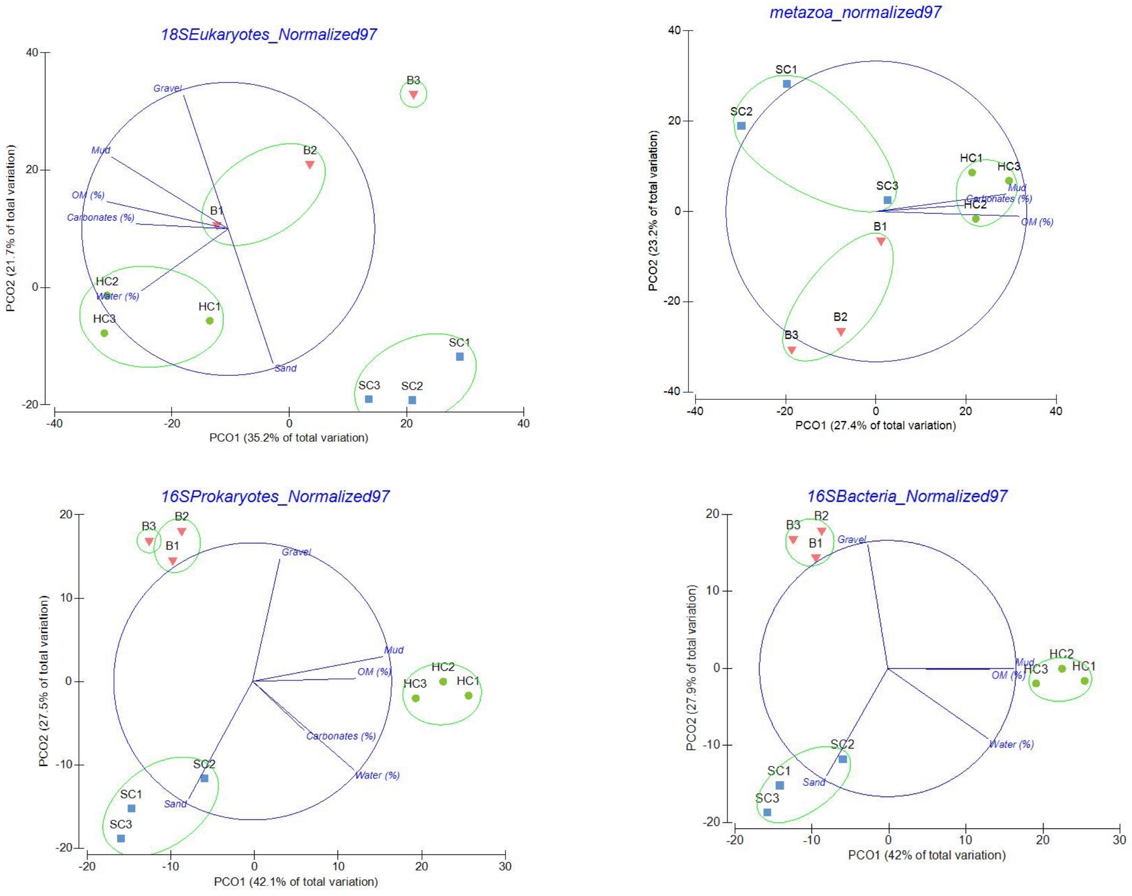

Supplementary Figure S3- Principal Coordinate analysis based on similarity profiles (green line > 70% similarity) on normalized datasets, for eukaryotes, prokaryotes, metazoan and bacteria. Analysis was based on Sorensen similarity coefficient using a presence-absence matrix. Environmental variables that showed the greatest individual effect (BIOENV, Pearson rho>0.45) on each phyla community structure are also represented (blue lines).

Fonseca VG, Kirse A, Hendrik G,Vause BJ, Drago T, Power DM, Peck LS and Clark MS. Metabarcoding the Antarctic Peninsula biodiversity: life under ice using a multi-gene approach.

Supplementary Information

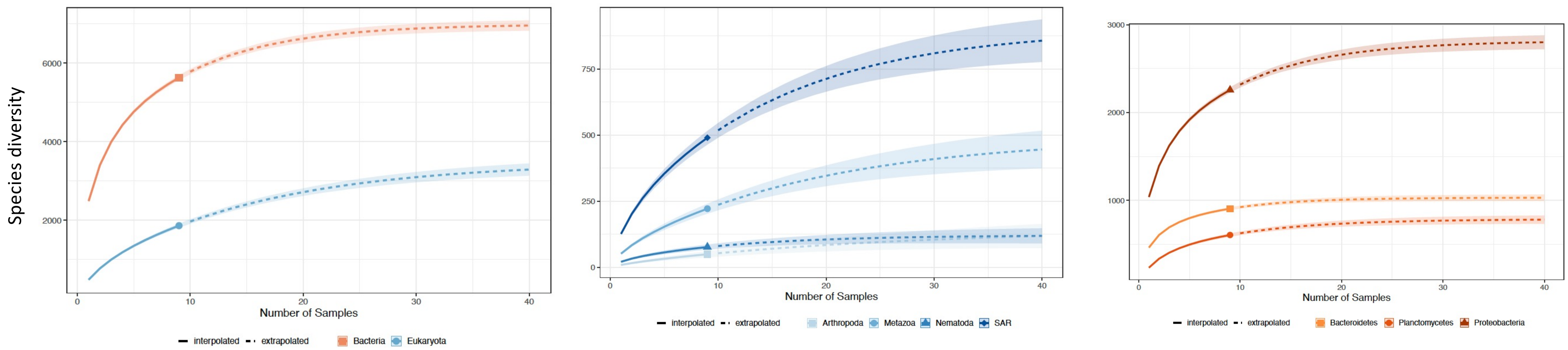

Supplementary Figure S4- Accumulation curves showing extrapolation of species diversity (Hill numbers) as a function of sample size, evidencing that the Antarctic microbial diversity is still to be discovered. Plots are shown for main groups such as Eukaryotes and Bacteria but also for other dominant phyla such as Arthropoda, Nematoda, Metazoa and SARS (b) and Proteobacteria, Planctomycetes and Bacteroidetes (C). Sample-size-based R/E curves with extrapolations of Hill numbers for presence-absence were estimated using R-package iNEXT at default settings (40 knots, 95% confidence intervals generated by the bootstrap procedure (50 bootstraps)).

Fonseca VG, Kirse A, Hendrik G,Vause BJ, Drago T, Power DM, Peck LS and Clark MS. Metabarcoding the Antarctic Peninsula biodiversity: life under ice using a multi-gene approach.

Supplementary Information

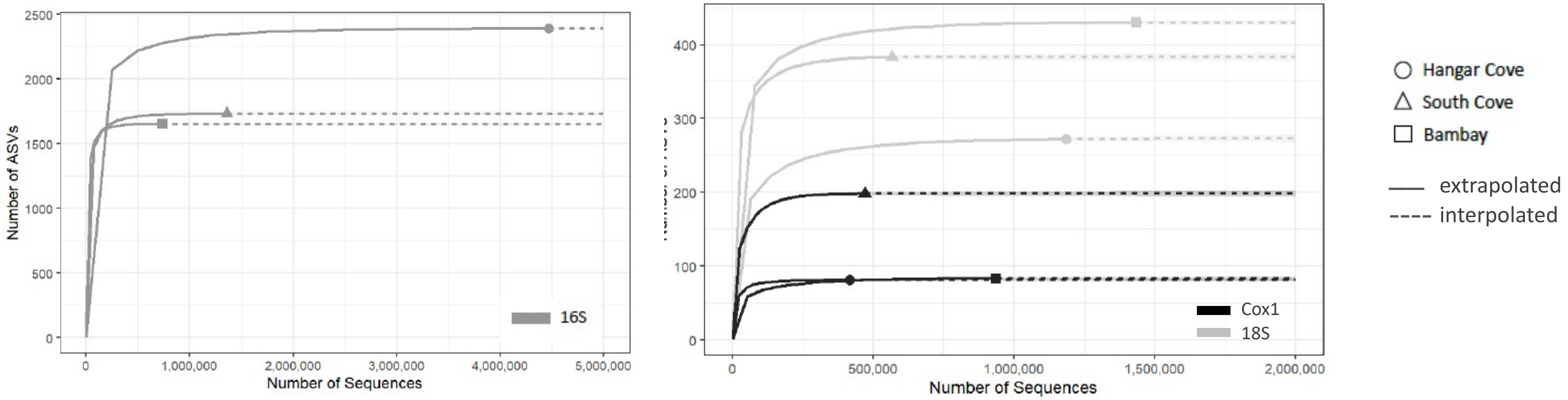

Supplementary Figure S5.1- Rarefaction curves showing the number of ASVs found (interpolated) and estimated (extrapolated) for the 16S gene and for the 18S and Cox1 genes as a function of number of sequences at 99% sequence identity threshold. Sequencing depth was sufficient for sampling site and marker.

Fonseca VG, Kirse A, Hendrik G,Vause BJ, Drago T, Power DM, Peck LS and Clark MS. Metabarcoding the Antarctic Peninsula biodiversity: life under ice using a multi-gene approach.

Supplementary Information

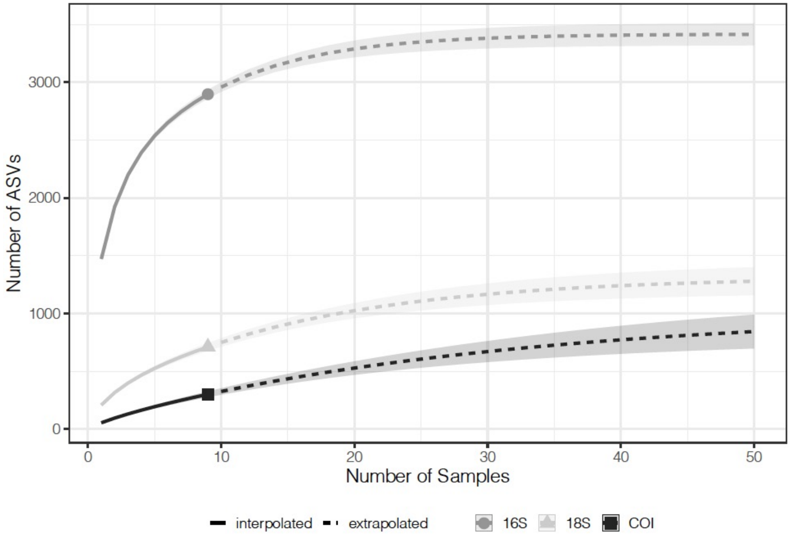

Supplementary Figure S5.2- Accumulation curves showing the number of ASVs found (interpolated) and estimated (extrapolated) as a function of number of samples (b) at 99% sequence identity threshold. Sampling effort was not enough to assess Antarctic benthic diversity.

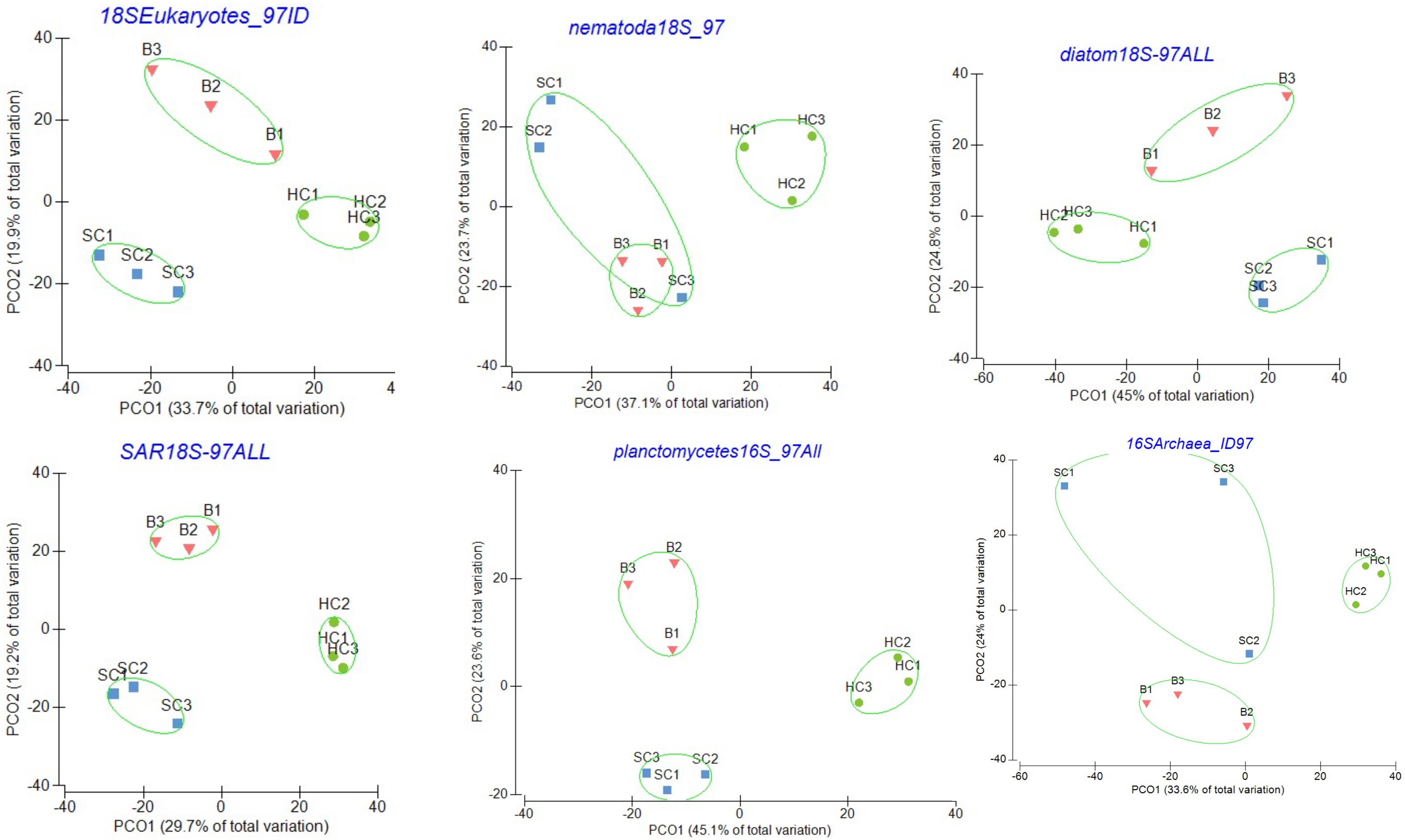

Supplementary Figure S6- Principal Coordinate analysis based on similarity profiles (green line > 70% similarity) for eukaryotes, nematodes, diatoms, SARs, planctomycetes bacteria and Archaea. Analysis was based on Sorensen similarity coefficient using a presence-absence matrix. Environmental variables that showed the greatest individual effect (BIOENV, Pearson rho>0.45) on each phyla community structure are also represented (blue lines).

Supplementary Information

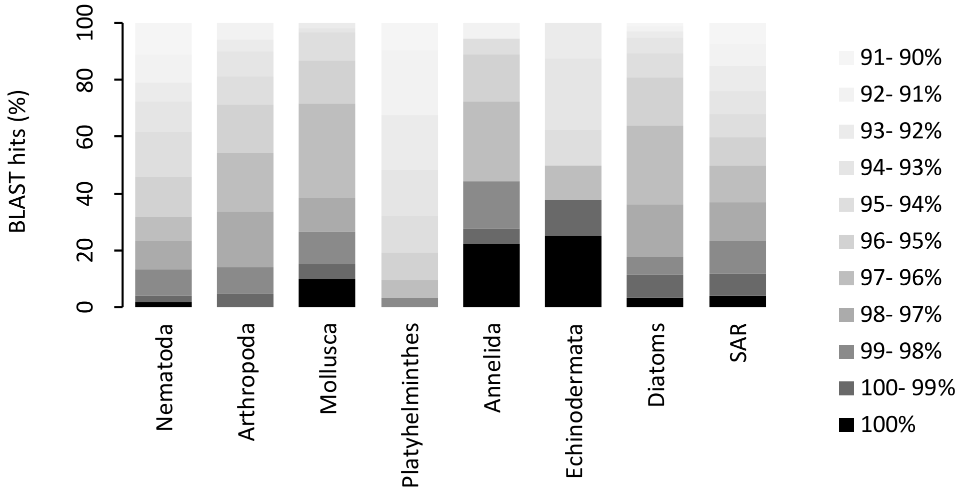

Supplementary Figure S7- Percentage identity BLAST match for each of the main eukaryote phyla against GenBank databases using the 18S rRNA gene. Colour gradients of black, dark grey to light grey indicate: proportion of ASVs with 100% blast matches, between 99-97% and bellow 96% up to 90% blast matches, respectively.
